# Methanogenesis from wastewater stimulated by addition of elemental manganese

**DOI:** 10.1038/srep12732

**Published:** 2015-08-05

**Authors:** Sen Qiao, Tian Tian, Benyu Qi, Jiti Zhou

**Affiliations:** 1 Key Laboratory of Industrial Ecology and Environmental Engineering (Ministry of Education, China), School of Environmental Science and Technology, Dalian University of Technology, Dalian 116024, P.R. China

## Abstract

This study presents a novel procedure for accelerating methanogenesis from wastewater by adding elemental manganese into the anaerobic digestion system. The results indicated that elemental manganese effectively enhanced both the methane yield and the production rate. Compared to the control test without elemental manganese, the total methane yield and production rate with 4 g/L manganese addition increased 3.4-fold (from 0.89 ± 0.03 to 2.99 ± 0.37 M/gVSS within 120 h) and 4.4-fold (from 6.2 ± 0.1 to 27.2 ± 2.2 mM/gVSS/h), respectively. Besides, more acetate consumption and less propionate generation were observed during the methanogenesis with manganese. Further studies demonstrated that the elemental manganese served as electron donors for the methanogenesis from carbon dioxide, and the final proportion of methane in the total generated gas with 4 g/L manganese addition reached 96.9%, which was 2.1-fold than that of the control (46.6%).

Comparing with the aerobic treatment, biological methanogenesis from wastewater under anaerobic conditions is widely recognized as a highly sustainable waste treatment process due to the conversion of wastewater’s organic content into renewable bioenergy in the form of methane rather than waste activated sludge^1^,^2^,^3^. Anaerobic digestion is a complex biological process, usually including three stages: hydrolysis-acidogenesis, acetogenesis and methanogenesis^4^,^5^. Specifically, the complex nutrient substance (primary polymers of carbohydrates and proteins) are converted to soluble organic compounds and further to soluble monomers, and subsequently the hydrolysis products were fermented to various intermediated products such as volatile fatty acids (VFAs), followed by the conversion of these VFAs into acetic acid, CO_2_ and H_2_ by acetogenic bacteria. Finally, methanogenic archaea utilize the acetic acid and H_2_ as substrates to produce methane. Though acetate as methanogenic substrate, H_2_ is also an important precursor to hydrogenotrophic methanogens, which can use H_2_ as electron donors for biosynthesis of methane by reducing CO_2_^6^. Furthermore, some studies recently found that syntrophic acetate oxidation (acetate is first oxidized to H_2_ and CO_2_) coupled to hydrogenotrophic methanogenesis was a dominant pathway in thermophilic methanogenic reactors^7^,^8^,^9^,^10^. Thus, methanogenesis facilitated by accelerating the activity of hydrogenotrophic methanogens and increasing the proportion of methane in the biogas had attracted much attention nowadays.

H_2_, the intermediate for methane synthesis, could be produced from organic wastes by biological fermentation, but yields were limited^11^. Consequently, in order to enhance the methane production rate, providing moderate amount of H_2_ seemed to be an efficient method. Recently, bioelectrochemical systems were widely utilized to assist microbial production of methane and hydrogen from wastewater and waste activated sludge^12^,^13^. Although the detailed mechanism of biological methanogenesis facilitated by electrochemical systems remained unclear, one proposed pathway of methane production was that the methanogens could utilize the abiotically produced H_2_, which obtained electrons from the cathode, to reduce the CO_2_, produced by acetate oxidation or graphite anode oxidation^14^. In addition, researchers found that elemental metals could serve as electron sources for biological methane formation from CO_2_ through cathodic depolarization^15^,^16^. Among all the metals, zero valent iron was the most efficient and widely-used in anaerobic biological waste treatment, such as wastewater and waste activated sludge, with the intention of enhancing the methanogenic performance^17^,^18^. A recent study by Feng *et al.* (2014) indicated that the addition of zero valent iron could enhance CH_4_ production rate by 43.5% in anaerobic digestion of waste activated sludge^19^. Besides acting as electron donors, zero valent iron had many other functions in the anaerobic systems due to its reductive properties, such as enhancing acidogenesis^20^ and acetogenesis^21^.

Manganese metal, a reductive material, offered many similar physicochemical properties compared to elemental iron. Lorowitz *et al.* previously demonstrated that methanogenesis by *Methanobacterium thermoautotrophicun* could occur coupled to the anaerobic oxidation of manganese and some other metals^16^. Though there were few studies on methanogenesis from wastewater with elemental manganese addition, it was assumed that the manganese metal could also serve as electron donors for methane formation from CO_2_ and be more effective than iron element due to its stronger reducibility.

Therefore, the main aim of the present study was to explore a simple and efficient method for accelerating the biological methanogenesis through adding elemental manganese into an anaerobic digestion system. To our best knowledge, it was the first time to enhance this process by elemental manganese addition. In order to evaluate the methanogenesis performance, methane production rates with different dosage of elemental manganese addition were measured, volatile fatty acids and pH with and without elemental manganese addition were determined, and the effects mechanism of elemental manganese on biological methanogenesis was also investigated.

## Results and Discussion

### Effect of Elemental Manganese on Methanogenesis

**Figure 1:**
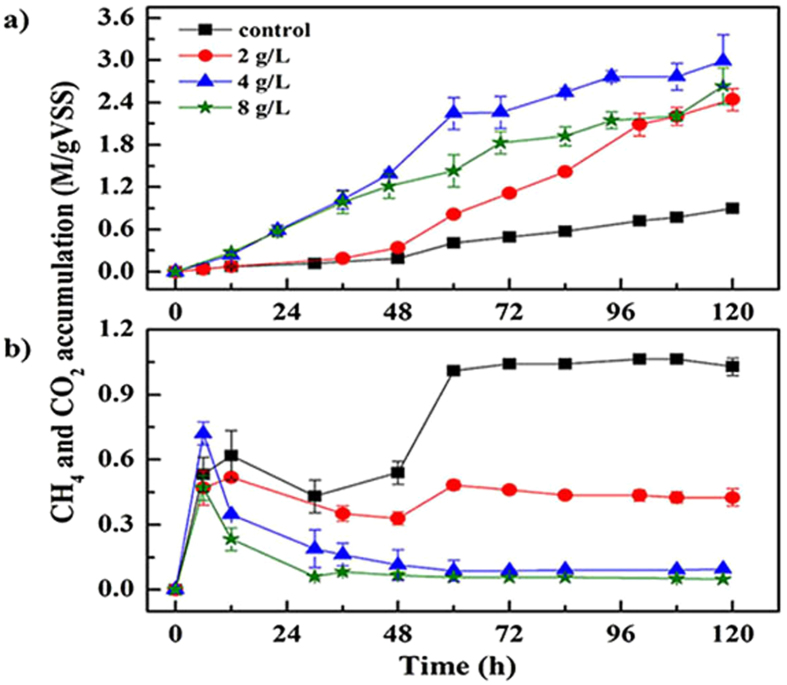
CH_4_ (**a**) and CO_2_ (**b**) production during the anaerobic digestion with different dosages of elemental manganese addition. Error bars represent standard deviations of triplicate tests.

During the batch experiment, glucose was utilized as organic carbon source, which could be fermented to VFAs rapidly. Therefore, the methanogenesis process was proved to be the rate-determining step^20^. Figure 1a described the methane accumulation in the head space within the whole operation of 120 h. The methane production rate of the control was slow before 48 h due to the accumulation of VFAs, exactly, the propionic acid, which could inhibit the activity of methanogenic archaea^22^. Then, after a period of adaptation, the methane production rate increased since 60 h and it was calculated as about 6.2 ± 0.1 mM/gVSS/h during the whole operation period. In order to investigate the effect of elemental manganese on the methanogenesis, 2 g/L, 4 g/L and 8 g/L of manganese powder were added into the anaerobic systems respectively. With 2 g/L manganese powder addition, the methane accumulation before 48 h slightly increased, and then a sharp rise was observed after 60 h with a final methane yield of 2.44 ± 0.15 M/gVSS and a production rate of 21.5 ± 1.5 mM/gVSS/h, which were about 2.7-fold and 3.5-fold higher than those of the control. An interesting result was obtained with 4 g/L and 8 g/L manganese powder additions that the methane producing process occurred rapidly without adaptation, compared with the other two experimental assays. In addition, both the methane yields and the production rates were significantly enhanced with final methane yields of 2.99 ± 0.37 and 2.64 ± 0.26 M/gVSS and production rates of 27.2 ± 2.2 and 21.5 ± 0.3 mM/gVSS/h, respectively. However, the increase in elemental manganese dosage to 8 g/L did not cause the highest methane yield and producing rate, which indicated that 4 g/L might be a more appropriate addition concentration for methanogenesis in the present study.

The CO_2_ concentrations were also detected during the whole research and the results were shown in Fig. 1b. The CO_2_ concentrations reached a peak in 12 hour and then it began to decrease mainly due to the beginning of methanogenesis process. As predicted, an obvious difference in the CO_2_ concentrations was observed with elemental manganese added. The consumption rates of CO_2_ with elemental manganese additions were much higher than that of the control. Moreover, after 46 hours, nearly no CO_2_ accumulations were determined with 4 g/L and 8 g/L elemental manganese added and a much lower accumulation was observed with 2 g/L elemental manganese added than that of the control, which showed a precipitate rise between 36 and 60 hours and then remained at a steady level. The difference of CO_2_ amount between the control and the 4 g/L elemental manganese assays was about 0.93 M/gVSS. Therefore, it was reasonable to consider that the additional CH_4_, generated with manganese added was converted from the CO_2_.

**Figure 2:**
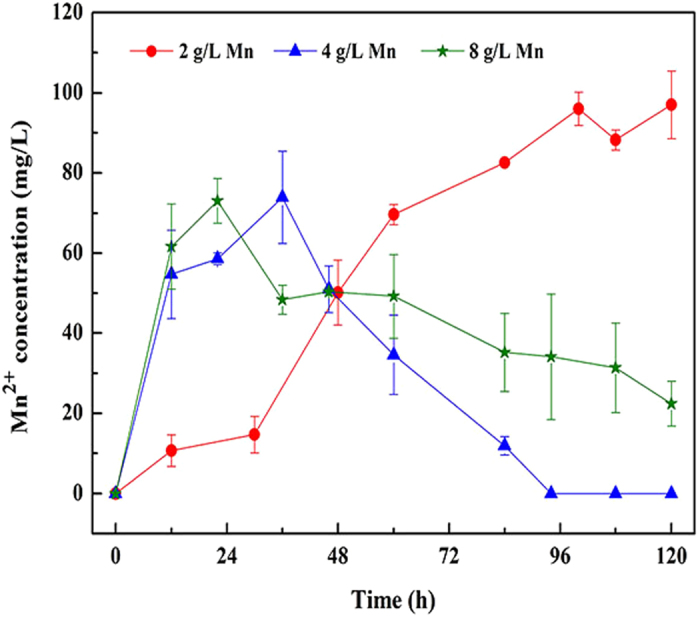
Manganous ions concentrations during the research operation. Error bars represent standard deviations of triplicate tests.

Electrons, generally provided by hydrogen, were needed to accomplish the conversion from CO_2_ into CH_4_ with hydrogen as substrate^23^. Literatures had shown that several kinds of elemental metals could serve as initial electron donors for CH_4_ formation from CO_2_ through cathodic depolarization^15^,^16^. In the present study, manganous ions in the liquid phase were determined to indicate whether the additional manganese could release electrons as donors for the methanogenesis and the results were shown in Fig. 2. The manganous ions concentration with 2 g/L manganese added increased slowly before 100 h and then became steady around 100 mg/L. However, as the concentrations of manganese increased to 4 and 8 g/L, the manganous ions production rates increased rapidly, and reached the peak about 75 mg/L at 36 h and 22 h, respectively. Then, the manganous ions concentration began to decrease. Particularly, it lowered to zero with 4 g/L manganese added at 96 h.

These results indicated that the additional manganese could act as electron donors, which was similar with other metals^15^. Since the anaerobic sludge system consisted mixed bacteria and it was assumed that some kinds of heterotrophic manganese-oxidizing microorganisms might exist in the anaerobic digestion system, which could oxidize the manganous ions to manganese dioxide, leading to a reduction in the manganous ions concentrations. Besides, another possibility could be direct electron transfer, in which methanogens functioned as manganese oxidizer, taking electrons directly from manganese to reduce CO_2_.

Moreover, careful examination about CH_4_ production rates and manganese ions concentrations showed that the CH_4_ production rate was significantly increased in the presence of manganese ions (Figs 1a and 2). Specifically, with 4 g/L and 8 g/L elemental manganese added, the concentration of manganese ions increased rapidly to the peak and maintain around 40 mg/L during the initial 60 hours. And in this duration, the CH4 production rates were particularly higher than that of the control, while with 2 g/L elemental manganese addition, the CH_4_ production rate did not raise sharply until the manganese ions in the liquid phase reached about 50 mg/L at 48 h. Subsequently, the CH_4_ production rates with 4 g/L and 8 g/L manganese dropped slightly with the decrease in the manganese ions, but with 2 g/L manganese added, the CH_4_ production rate increased constantly due to the presence of high-dosage of manganese ions. This reasonable correlation between CH_4_ production rate and manganese ions implied that the generated manganese ions should be a reliable reason for the enhancement in methanogenesis, which was consistent with a previous research reported by Nandan *et al.*^24^.

**Figure 3:**
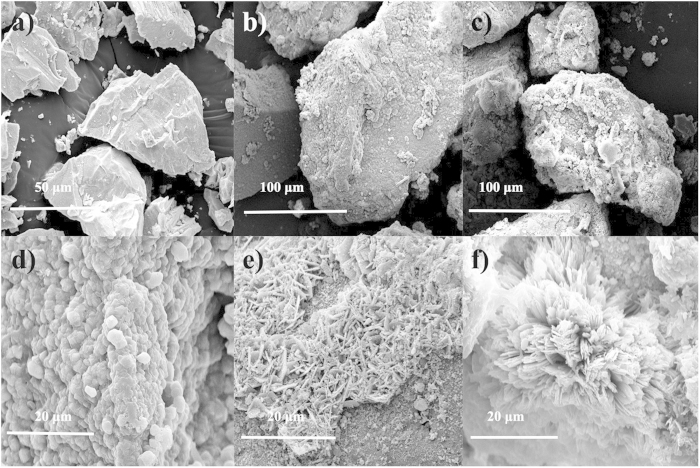
SEM images of (**a**) initial manganese power, (**b**,**c**) final manganese power, (**d**) ball-like MnO_x_, (**e**) rod-like MnO_x_, (**f**) flower-like MnO_x_.

Scanning electron microscopy (SEM) analysis was conducted using a FEI Company’s Quanta 450 field emission scanning electron microscope to verify the possible generated manganese oxides based on such hypothesis. Figure 3 showed the SEM observation of the manganese powder and the generated manganese oxides. The surface of the initial added manganese power were flat with sharp edges (Fig. 3a), while after 120 h of anaerobic reaction, the surface turned to be rough with a layer of manganese oxides distribution (Fig. 3b,c). It should also be noted that the generated manganese oxides possessed a variety of structure. Figure 3d showed a kind of ball-like manganese oxides with an average diameter about 3 μm and Fig. 3e depicted a kind of rod-like manganese oxide with an average length about 5 μm. Both of these two kinds of manganese oxides were common in the manganese oxidation process^25^. Interestingly, this study obtained a kind of flower-like manganese oxides (Fig. 3f), which was recognized to be a new type of biogenic manganese oxides.

The observation of manganese oxides indicated the existence of manganese-oxidizing process in the anaerobic digestion system. In order to investigate the functional strains during the manganese ion oxidation, clone library analysis of 16S rRNA genes of the seed inoculum (Bacteria) was conducted. The results indicated that *Firmicutes*, a kind of well-known manganese-oxidizing bacteria^26^,^27^, was present in the anaerobic sludge samples (Supplementary Information), which was proposed to be the functional strains that participated in the oxidation of manganese ions.

These results showed that appropriate dosages of manganese addition could not only enhance the methanogenesis process, but also avoid the potential pollution that caused by manganous ions^28^. Moreover, many literatures had demonstrated that the biogenic manganese oxides were highly reactive minerals, which offered a much higher sorption and oxidation capacities for metal ions than abiotic ones^29^. Therefore, enhancement of methanogenesis process by elemental manganese addition seemed to be an efficient and economic method due to the recovery of both the biofuel and the biogenic manganese oxides with little pollution.

The pH value was an important influence factor for biological methanogenesis. The initial pH values of the anaerobic system were adjusted to 7.2 ± 0.1 with 0.1 M HCl and the final pH values were 5.5 ± 0.1 (control), 7.4 ± 0.1 (2 g/L), 8.4 ± 0.1 (4 g/L) and 8.9 ± 0.1 (8 g/L), respectively, presenting an obvious increase trend with higher elemental manganese levels. During the methanogenesis process, the pH values could increase due to the consumption of protons, which were utilized to form H_2_, the precursor for hydrogenotrophic methanogenesis^23^,^30^. The higher pH values obtained with elemental manganese added was closely related with the methane yields, which might indicate that the production of H_2_ as an intermediate and further accelerating methanogenesis was reasonable.

### Methane production by microbiological catalysis

Although excess CH_4_ was determined with CO_2_ consumption, it was not ascertained whether the conversion was mediated by microbiological process. For such reasons, two kinds of methods were utilized to verify whether the excess CH_4_ production process was catalyzed by microorganisms.

**Figure 4:**
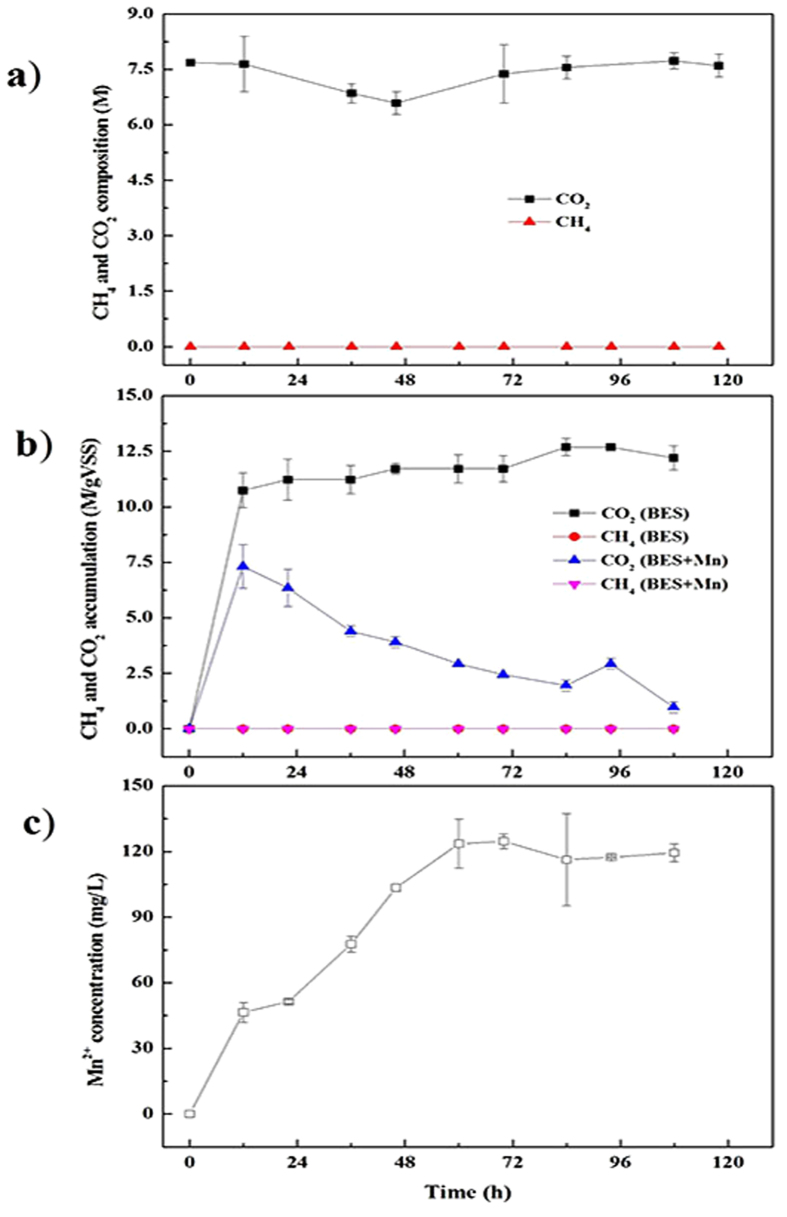
Gas composition without anaerobic sludge supplement (**a**) and with BES addition (**b**) and Mn^2+^ concentration with BES addition (**c**). Error bars represent standard deviations of triplicate tests.

First, the anaerobic sludge was removed from the digestion system and other conditions were kept the same as the batch experiment with 4 g/L manganese added. Figure 4a described the gas composition during the whole operation without anaerobic sludge supplement. It was obviously that no CH_4_ was determined during the whole operation period, though the CO_2_ decreased to 6.59 ± 0.31 M at 46 h. Moreover, a much higher level of manganous ions of 415.0 mg/L was observed in the end. It was reasonably considered that the high CO_2_ pressure in the gas phase resulted in an increase in the bicarbonate ion in the liquid phase, which accelerated the cathodic depolarization of elemental manganese. Furthermore, the created manganous ions could not be further oxidized in the absence of the manganese-oxidizing microorganisms. Therefore, the manganous ions concentration in this test was higher than that of the batch experiments. Besides, due to the lack of methanogens in the anaerobic system, the consumed CO_2_ could not be utilized to synthetize CH_4_, though electrons could be provided by the elemental manganese. These results indicated that the simultaneous consumption of CO_2_ and manganese was mediated by chemical reaction and microorganisms were required to participate in the CH_4_ production process.

In addition, since the digestion medium was prepared without sterilization, the organic contents could be converted into CO_2_ in the presence of microorganisms, which might cause the increase in the CO_2_ contents after 46 h. The high manganous ions concentration obtained in this test could also imply the existence of manganese-oxidizing microorganisms in the anaerobic sludge.

Secondly, one kind of methanogenesis inhibitor, 2-bromoethanesulfonate (BES) was added into the medium with complete digestion system containing sludge. 4 g/L manganese was supplied into the system and the test was carried out under the same conditions as mentioned above except for addition of 20 mM BES.

Both CH_4_ and CO_2_ productions with BES added were shown in Fig. 4b. CO_2_ was rapidly generated before 12 h by the acidogenesis and acetogenesis processes. A sudden decrease trend in CO_2_ was detected with manganese added, while it showed a slow increase trend in the absence of manganese. Though manganous ions were also determined with BES added (Fig. 4c), there was no CH_4_ produced due to the inhibitory effects on the microbiological methanogenesis caused by BES. Thus, the resluts could further indicate that the excess CH_4_ generated in the bacth experiment was mediated by microbiological catalysis.

### Methanogenesis from CO_2_ with manganese as electron donors

**Figure 5:**
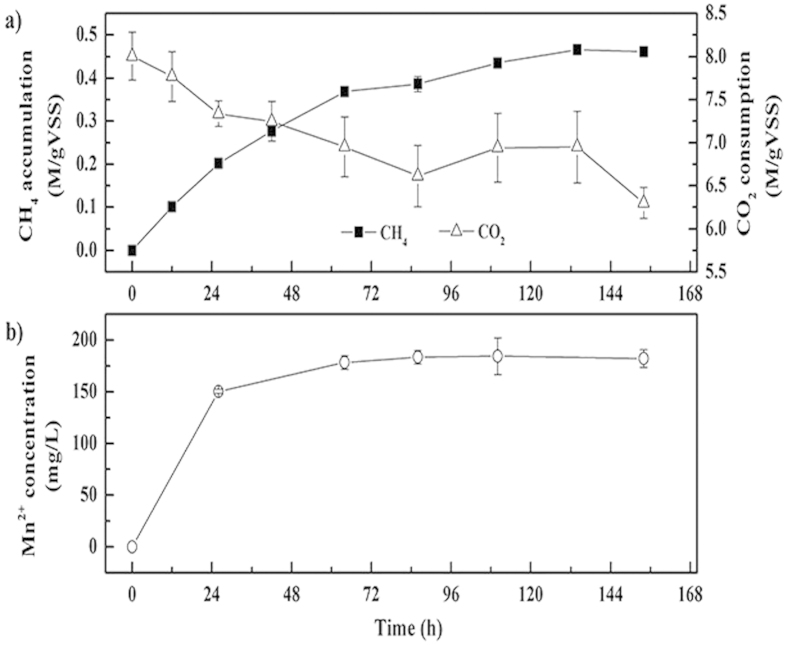
CH_4_ production and CO_2_ consumption (**a**) and Mn^2+^ concentration (**b**) with CO_2_ as the sole carbon source. Error bars represent standard deviations of triplicate tests.

As mentioned above, the excess generated methane in the anaerobic systems was assumed to be converted from CO_2_ with elemental manganese as electron donors on the basis of the simultaneous CO_2_ and manganese consumptions and CH_4_ and manganous ions productions. In order to confirm this assumption, another test was carried out with CO_2_ as the sole carbon source in the anaerobic mixture and a manganese concentration of 4 g/L. Figure 5a depicted the CH_4_ production and CO_2_ consumption in the gaseous phase. The final CH_4_ accumulation reached about 0.46 ± 0.01 M/gVSS, which was almost equivalent to the actual loss in CO_2_ (the actual loss in CO_2_ was about 0.48 ± 0.02 M/gVSS and the excess consumption of CO_2_ in Fig. 5a was caused by multiple sampling of gas components from the head space). As expected, manganous ions were also detected (Fig. 5b) and no decrease trend in the manganous ions concentration was found, which was consistent with the preceding discussion, due to the short of organic carbon sources for the heterotrophic manganese-oxidizing microorganisms. Consequently, it was proved that the loss of CO_2_ in the batch experiments with manganese added was utilized by methanogens to create CH_4_ with electrons supplied by elemental manganese through cathodic depolarization. Thus the whole methanogenesis process with manganese addition could be represented as the following reaction:


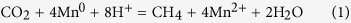


### Effects of Elemental Manganese on VFAs generation

**Figure 6:**
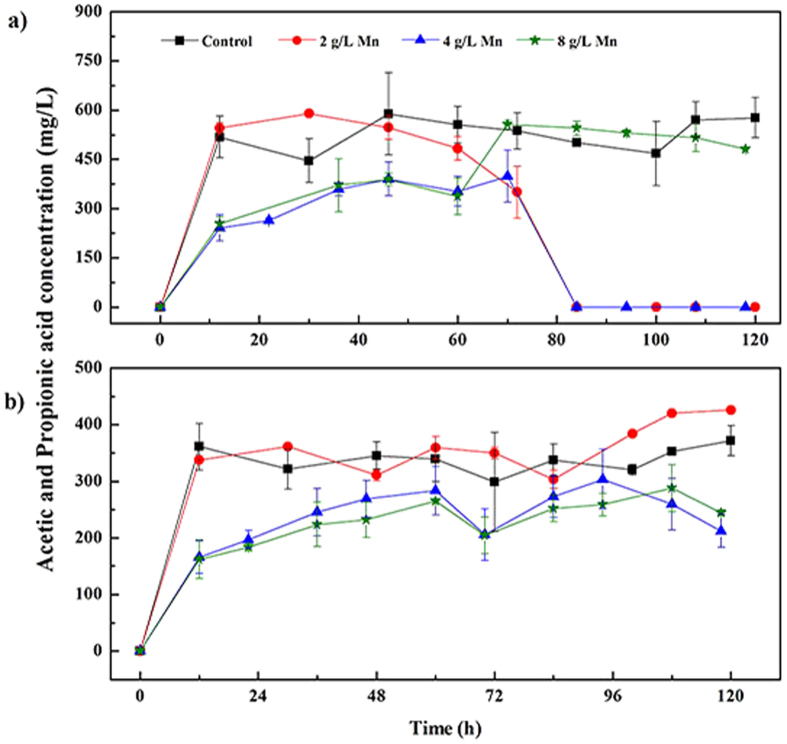
Comparison of (**a**) acetic acid and (**b**) propionic acid concentrations with different dosages of elemental manganese addition. Error bars represent standard deviations of triplicate tests.

VFAs concentrations were determined during the whole operation period and only acetate and propionate were detected in the liquid phase. The production and consumption of these two kinds of VFAs were shown in Fig. 6. Simple organics could be rapidly fermented to volatile acids under anaerobic conditions^18^, thus, both the acetate and propionate were produced within 12 hours with glucose served as substrates. With manganese addition, significant differences in acetate concentrations were observed in the present study (Fig. 6a). The acetate concentrations of control fluctuated between 450 mg/L and 600 mg/L and the small amount consumption of acetate, one of the substrates for methanogens, was consistent with the CH_4_ production during the research (Fig. 1a). However, with 2 g/L manganese added, the acetate concentration dropped to 0 mg/L at 84 h. As a result, the CH_4_ production rate increased distinctly during this period (Fig. 1a). The increase in the manganese concentration to 4 g/L resulted in a reduction in the acetate at 12 h, which was only about 250 mg/L, just 45.5% of the control. Besides, it was similar with the former that the acetate concentration decreased to 0 mg/L at 84 h. The acetate concentration with 8 g/L manganese added changed intricately. Though a reduction in acetate generation was also found at 12 h, the concentration increased suddenly at 60 h after a period of stationary phase, and then it declined slowly. It was assumed that some inhibitory effects on the methanogenesis occurred in the digestion system.

As mentioned above, additions of 4 and 8 g/L elemental manganese could stimulate methanogenesis from CO_2_. And further calculation of CH_4_ yield and CO_2_ consumption showed that part of CH_4_ was produced through other pathway, exactly, by the aceticlastic methanogens using acetate as substrate. Therefore, both hydrogenotrophic methanogens and aceticlastic methanogens activities were enhanced by additions of manganese. Meanwhile, careful examination and comparison of CH_4_ production and acetate consumption showed that after 84 h, CH_4_ yield with 2 and 4 g/L manganese added still showed increase trend (Fig. 1a), although acetate was consumed entirely (Fig. 6a). Moreover, the CH_4_ production rate with 4 g/L manganese added was lower than that with 2 g/L manganese added during this period, which might be caused by CO_2_ limitation (Fig. 1b). However, the CH_4_ production did not showed a higher increase trend with 8 g/L manganese, though high acetate level was observed. According to Fukuzaki *et al.* that methanogenesis from acetate could proceed well from pH 6 to pH 8^31^, thus, the high pH value (8.9 ± 0.1) caused by manganese addition was assumed to be a reasonable explanation for acetate accumulation at high manganese dosing.

Nevertheless, the effect of manganese on the propionate production seemed to be relatively simple. Propionate concentrations with 4 and 8 g/L manganese were quite different from those of the control and with 2 g/L manganese addition, which were about 42.8% of the latter two. The results in VFAs concentrations demonstrated that the added manganese could also enhanced the acetoclastic methanogenesis.

## Methods

### Bacterial Inoculum and Medium

The anaerobic sludge utilized as initial inoculum was collected from a local domestic sludge treatment plant in Dalian. A synthetic wastewater consisting of (g/L) the following: peptone 0.1, yeast extract 0.01, NaHCO_3_ 0.075, KH_2_PO_4_ 0.0225, NaCl 0.05, CaCl_2_ 0.025, MgSO_4_·7H_2_O 0.075, FeSO_4_·7H_2_O 0.018, and 1.25 mL/L of trace element solution, which containing (g/L) EDTA·Na_2_ 15, ZnSO_4_·7H_2_O, CuSO_4_·5H_2_O 0.15, CoCl_2_·6H_2_O 0.24, MnCl_2_·4H_2_O 0.99, NaWO_4_·2H_2_O 0.05, NaMoO_4_·2H_2_O 0.22, NiCl_2_·6H_2_O 0.19, NaSeO_4_·10H_2_O 0.21 and H_3_BO_4_ 0.014 was used throughout the study. Glucose and carbon dioxide were used as carbon sources according to different requirements. Elemental manganese power (M105838, 99.99% metals basis) was bought from Aladdin^@^.

### Experimental Procedure

Investigation of the effects of elemental manganese on methanogenesis was carried out through batch experiments with different manganese concentrations (0, 2, 4, and 8 g/L). The manganese powder was added into the anaerobic digestion system at the beginning with an aliquot (50 mL) of the synthetic medium, which contained 1 g wet weight sludge (MLVSS of 1.8 g/L) and a chemical oxygen demand (COD) of 1.0 g/L with glucose as carbon source. The fermentation system was purged with dinitrogen gas for 10 minutes to removal dissolved oxygen in the liquid phase and air in the head space. The initial pH of the medium was adjusted to 7.2 ± 0.1 with 0.1 M HCl and the temperature was maintained at 35 ± 1 °C in a water bath shaker at a shaking speed of 150 rpm to keep full contact between the sludge and the medium. The samples were collected regularly using a sterile syringe for the aqueous samples and a gastight syringe for the gaseous samples, respectively.

Afterwards, in order to verify that the enhanced methane production with elemental manganese added was achieved by microbial catalysis, two sets of batch experiments were performed. In the first set of experiments, no anaerobic sludge was supplied into the digestion system and the system consisted of 50 ml synthetic medium and 4 g/L elemental manganese powder. Besides, a certain concentration of CO_2_ was aerated into the system to investigate the potential conversion of CO_2_ into CH_4_ under such conditions. The experiments lasted for 118 hours and gaseous samples in the headspace were collected with a gastight syringe punctually for the determination of CH_4_. Subsequently, in the other set, 2-bromoethanesulfonate (BES), a kind of widely used methanogenesis inhibitor was added into the digestion system for further investigation. The digestion system contained 50 mL synthetic medium with 1.8 g/L MLVSS and 20 mM BES and glucose (1.0 gCOD/L) was used as carbon source during this test. 4 g/L manganese was added into the system with the intension of studying the function of manganese during the digestion process. Systems with no manganese addition was designed as control. The experiments lasted for 108 hours under the same conditions as mentioned above.

Finally, to study the feasibility of methanogenesis from CO_2_ with elemental manganese added, test with CO_2_ as sole carbon source was conducted. The anaerobic digestion system contained 50 ml synthetic medium and 44.4 M CO_2_. 1 g wet weight sludge (MLVSS of 1.8 g/L) and 4 g /L elemental manganese were added into the system, respectively. The test lasted for 146 hours and was operated under the same conditions as mentioned above. All the data shown in the present study were the mean values of triplicate experiments.

### Measurement of Methane and Carbon Dioxide Gases

Gases (methane and carbon dioxide) accumulated in the head space were periodically analyzed. 0.5 mL of gas was taken from the vial head space with a gastight syringe and directly injected into a gas chromatography (Techcomp, GC7900, China) with a thermal conductivity detector (TCD) and a stainless steel column (TDX-01, 4 mm × 2 m). Helium gas was used as the carrier gas with a flow rate of 30 mL min^−1^. The oven was set at 100 °C and the injector and detector were maintained at 100 and 120 °C, respectively. Standard curves of each measured gas were established by injecting a known volume of high purity standard gas into the GC.

### Analyses of Mn (II) and Volatile Fatty Acid in the Aqueous Phase

Oxidized manganese, Mn (II) immediately produced in the aqueous phase, treated as a key factor investigating the oxidation of elemental manganese was quantified spectrophotometrically using the formaldoxime methods^32^ (Mn (II) or even Mn (III) would be oxidized to Mn (IV) under alkaline condition) with a UV spectrophotometer (V-560 UV/VIS Spectrophotometer, Jasco, Japan). The concentrations of volatile fatty acids (VFAs) were determined using a high performance liquid chromatography (Shimadzu, LC-20AT, Japan), equipped with an elite Hypersil ODS2 C18 column (25 μm, 4.6 × 250 mm) for separation at 40 °C and a diode array detector (SPD-M20A, Japan) for measurement at 210 nm. The mobile phase consisted of methanol (15%, V/V) and ultrapure water (pH 3.0, 85%, V/V) at a flow rate of 1 mL/min.

### Clone library analysis of 16S rRNA genes

Clone libraries were constructed from samples (Bacteria) of the seed inoculum. Bacterial 16S rRNA genes were amplified with forward primer 27F (5′-AGAGTTTGATCCTGGCTCAG-3′) and reverse primer 1392R (5′-ACGGGCGGTGTGTRC-3′)^33^. PCR was performed under the following conditions: 95 °C/5 min denaturation step; 35 cycles each of 95 °C/45 s, 50 °C/45 s, 72 °C/90 s; and a final extension step at 72 °C/10 min.

Triplicate PCR products were pooled and purified with Qiaquick PCR Gel Extraction Kit (QIAGEN, Stanford, CA, USA), cloned using the pMDTM18-T Vector System (TaKaRa, Dalian, China) with TOP10 competent Escherichia coli cells (Tiangen, Beijing, China) and plated on LB (Luria-Bertani) plates supplied with ampicillin (Sigma). Colonies were randomly picked, cultured overnight in LB broth supplemented with ampicillin, and then sequenced on both strands using the vector primers M13F-47 and M13R-48 in Invitrogen Inc. (Beijing, China). The nucleotide sequences were compared with those in GenBank database using nucleotide BLAST.

## Additional Information

**How to cite this article**: Qiao, S. *et al.* Methanogenesis from wastewater stimulated by addition of elemental manganese. *Sci. Rep.* **5**, 12732; doi: 10.1038/srep12732 (2015).

## Supplementary Material

Supplementary Information
