# Supplementary material for: Methanogenesis from wastewater stimulated by addition of elemental manganese
Source: Sci Rep. 2015 Aug 5;5:12732. doi: 10.1038/srep12732 (PMC4525485; doi:10.1038/srep12732)
Supplement: Supplementary Information [file srep12732-s1.pdf]

Title: Methanogenesis from wastewater stimulated by addition of elemental manganese

Sen Qiao*, Tian Tian, Benyu Qi, Jiti Zhou

Supplementary
Results of clone library analysis of 16S rRNA

Table 1 Closest phylogenetic affiliation of 37 clones of seed sludge based on BLAST comparison in the GenBank database.

No. of clones	Database access number	Closest related sequences	Similarity (%)
5	DQ129245	Uncultured bacterium	100
3	DQ168847	Uncultured Bacteroides sp.	100
2	NR041464	Parabacteroides johnsonii	99
1	DQ168843	Uncultured Olsenella sp.	99
3	AB742072	Uncultured Firmicutes bacterium	99
1	CU921886	Uncultured Firmicutes bacterium	99
2	EU236242	Uncultured Firmicutes bacterium	99
2	JX104019	Prevotella sp. S4-8	99
4	NR113098	Prevotella buccalis	99
1	EU639425	Ethanoligenens harbinense	99
3	KJ660075	Lactocossus lactis	100
3	AB678394	Clostridium beijerinckii	99
2	NR121710	Clostridium saccharobutylicum	99
3	NR042704	Desulfovibrio marrakechensis	99
2	AY188845	Clostridium sp.	99

Experimental

No. of clones	Database access number	Closest related sequences	Similarity (%)
